# The mechanical principles behind the golden ratio distribution of veins in plant leaves

**DOI:** 10.1038/s41598-018-31763-1

**Published:** 2018-09-14

**Authors:** Zhi Sun, Tianchen Cui, Yichao Zhu, Weisheng Zhang, Shanshan Shi, Shan Tang, Zongliang Du, Chang Liu, Ronghua Cui, Hongjie Chen, Xu Guo

**Affiliations:** 1 State Key Laboratory of Structural Analysis for Industrial Equipment, Department of Engineering Mechanics, International Research Center for Computational Mechanics, Dalian University of Technology, Dalian, 116023 P. R. China; 2 Institute of Traffic and Transportation Engineering, Dalian Jiaotong University, Dalian, 116028 P. R. China

## Abstract

Tree leaves are commonly composed of thin mesophyll, carrying out photosynthesis under sunlight, and thick veins. Although the role of leaf veins in water transportation has been known for a long time, their role in providing structural support and guaranteeing large sunlighted area was rarely studied and remains elusive. Here, with use of a novel inverse optimization approach, we aim for uncovering the material design principle behind the unique pattern of venation. It is intriguing to observe that an almost Golden Ratio (GR) distribution of leaf veins always provides optimized structural behavior. Specifically, our research reveals, for the first time, that this unique GR distribution of relatively strong vein material is helpful for maximizing the bending stiffness and leading to a large sunlighted area which is vital for the photosynthesis process of a leaf. Moreover, the GR distribution of leaf veins is also observed in a wide class of plant leaf geometries (i.e., shape, thickness), where experimental evidence is provided for the optimized results. Therefore, our findings can not only serve to explain the mystery of veins GR distribution but also provide widely applicable guidelines on designing soft structures with exceptional mechanical performances.

## Introduction

Tree leaves are generally formed by two components: mesophylls and veins. The law for the fractal distribution of veins among mesophylls has arisen massive curiosity among scientists. For decades, great progress has been made, especially in the understanding of the role played by leaf veins in hydraulic transportation during plant growth^1–3^, which further triggers the development of medicine, agronomy, and hydraulic system^2–5^. In contrast, the mechanical functions of leave veins were barely discussed in literature, and most of the studies in record focus on the growing and drying process of leaves^6–8^.

**Figure 1:**
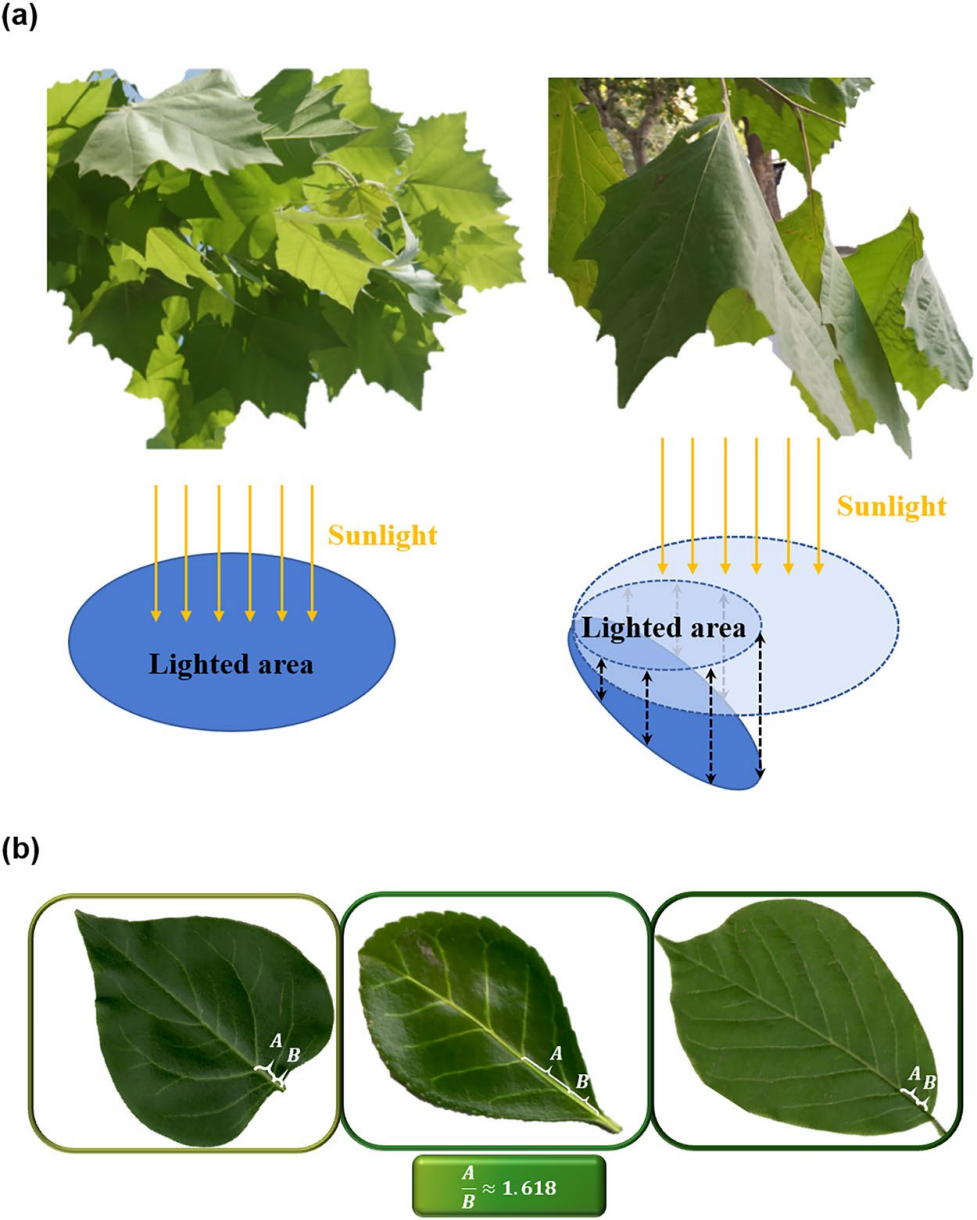
(**a**) Unfurling leaves and drooping leaves; representing the changes in sunlighted area. (**b**) Golden ratio in typical leaves.

As the plants’ principal agents of photosynthesis, unfurling leaves are expected to spread themselves so as to maximize the sunlighted area (Fig. 1a), and this is supposed to be achieved by utilizing the distribution of veins, which can be considered as the structural reinforcing components of leaves. In this report, the mechanical role of the fractal distribution of veins is investigated from a viewpoint of structural optimization. In order to capture the complex configuration of venation, we adopt the method of topological optimization, which can be used to find the optimized material distribution within a given design space under certain prescribed constraints. This method has been successfully applied for understanding the intriguing geometry of biological tissues^9–17^. The problem of searching the best venation can be translated to finding the optimized layout of the structural reinforcements that maximizes the structural stiffness of a leaf under a self-weight pressure load on its top surface. A very interesting observation from our topology optimization results is that, the optimized lengths of the first two sections on the main vein display a golden-ratio relationship, and this phenomenon has been widely observed in various ovate-shape leaves^18^, as shown in Fig. 1b.

The effect of leaf thickness and normalized bending stiffness on optimized venation is firstly investigated. Optimized venations of leaves with various normalized bending stiffness are examined to clarify the role of fractal veins on structural performance of leaves. The present study aims for providing insight into how plant may achieve more sunlighted area and better structural performance, which can be useful for the design of biomimetic functional structures.

## Results

### Bending stiffness of leaf

In conjunction with the geometric measurements of leaves recorded in literature^19,20^, the bending stiffness of mesophyll and vein can be calculated. Some parameters that characterize the geometrical and mechanical properties of typical leaves are listed in Table 1. For simplicity, the bending stiffness of vein reinforcements is normalized as 1, and the bending stiffness of leaf mesophyll is normalized as ${{\rm{10}}}^{-9}$ to ${{\rm{10}}}^{-3}$, in order to cover the full range of relative bending stiffness.

**Table 1:** Stiffness and geometry properties of typical leaves

Properties (unit)	Range
Young’s modulus of leaf mesophyll (GPa)	0.040^19^ – 2.15^20^
Young’s modulus of leaf vein (GPa)	0.099^19^ – 23.0^20^
leaf thickness (μm)	90.0^20^ – 430^19^
leaf vein thickness (μm)	622^20^ – 6420
Bending stiffness of leaf mesophyll (10^−6^ N*m)	2.67–15.6 × 10^3^
Bending stiffness of leaf vein (10^−6^ N*m)	2180–557 × 10^6^

### Loading condition

As shown in Fig. 1a, tree leaves tend to unfurl themselves in order to achieve a maximum sunlighted area. This is in contrast to the dehydrated drooping leaves, which are no longer self-supportive as their structural stiffness has been greatly reduced. Therefore, the photosynthesis efficiency is highly related to the structural deformation of a leaf under its self-weight. In this study, a uniform pressure load is applied on the surface of a leaf so as to mimic the effect induced by its self weight. It is assumed that the veins will not change the local density and self-weight pressure load.

### Optimized venation of ovate-shape leaves

**Figure 2:**
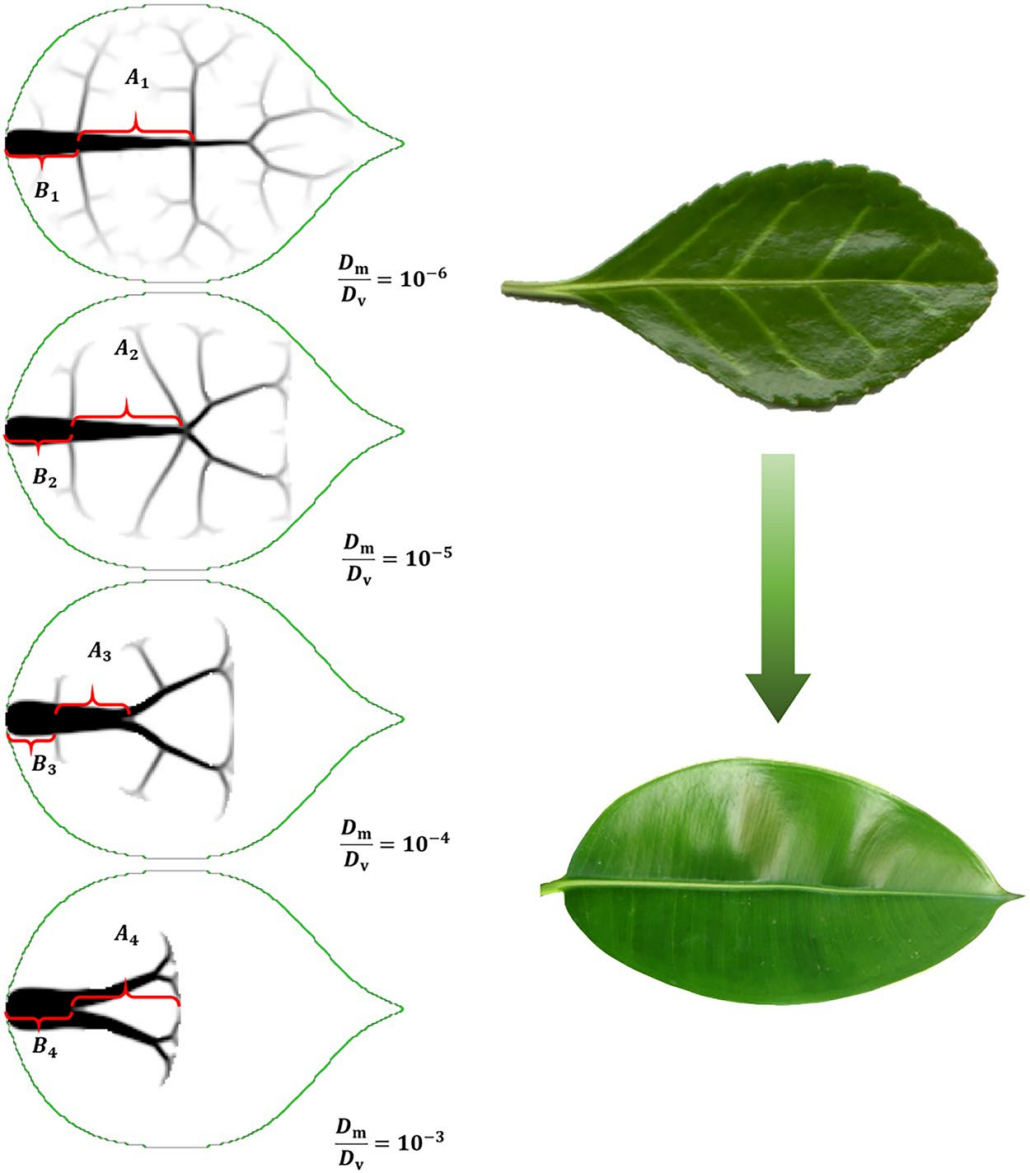
Comparison between optimized venations and actual veins; showing agreement in the influence of relative bending stiffness.

The structural role played by the fractal venation in supporting the ovate-shape leaves is firstly discussed. Figure 2 gives the optimized venation for ovate-shape leaves with different normalized stiffness. As described above, the optimization is carried out with 120 × 120 grid meshes and a vein area constraint of 5%. If we mark the first and second sections of the main vein as ${A}_{{\rm{n}}}$ and ${B}_{{\rm{n}}}$, respectively, the corresponding computational results are listed in Table 2. It is noted that the values of the section lengths appearing in this article are normalized by the total length of the main vein from which ${A}_{{\rm{n}}}$ and ${B}_{{\rm{n}}}$ are taken. In particular, the ${A}_{{\rm{n}}}$-to-${B}_{{\rm{n}}}$ ratio is calculated to be 1.6192, 1.6219, 1.6187 and 1.6182 with the relative stiffness ratio being ${{\rm{10}}}^{-6}$, ${{\rm{10}}}^{-5}$, ${{\rm{10}}}^{-4}$ and ${{\rm{10}}}^{-3}$, respectivy. The optimized venations and values of ${A}_{{\rm{n}}}/{B}_{{\rm{n}}}$ for leaves with relative stiffness ratio being ${{\rm{10}}}^{-9}$, ${{\rm{10}}}^{-8}$ and ${{\rm{10}}}^{-7}$ are not repeatedly given, since they are all mostly the same as those of ${{\rm{10}}}^{-6}$. It is likely to conclude that with various normalized stiffness values, all values of ${A}_{{\rm{n}}}/{B}_{{\rm{n}}}$ approximately take a Golden Ratio (GR) relationship in the optimized distribution of vein reinforcement that maximizes the structural stiffness of plate. This interesting finding provides a rationale, from a mechanical viewpoint, to the GR relationship widely observed in the venation of plant leaves. The GR venation can give rise to a higher structural bending stiffness (given prescribed areas of reinforcing materials), so as to maximize the sunlighted area.

**Table 2:** Normalized length of the first and second sections of main vein.

Relative bending stiffness ${{\boldsymbol{D}}}_{{\bf{m}}}/{{\boldsymbol{D}}}_{{\bf{v}}}$	Normalized length of the first section of main vein ${{\boldsymbol{B}}}_{{\bf{n}}}$	Normalized length of the second section of main vein ${{\boldsymbol{A}}}_{{\bf{n}}}$	Length ratio ${{\boldsymbol{A}}}_{{\bf{n}}}/{{\boldsymbol{B}}}_{{\bf{n}}}$
10^−6^	0.1794	05	1.6192
10^−5^	0.1698	0.2754	1.6219
10^−4^	0.1167	0.1889	1.6187
10^−3^	0.1658	0.2683	1.6182

Another interesting observation from the topology optimization results shown in Fig. 2 is that the stiffer the mesophyll is, the more concentrated the main vein becomes. For example, when the relative stiffness ratio is ${{\rm{10}}}^{-6}$, the topologically optimized distribution (on the top left of Fig. 2) consists of one main vein, three pairs of branch veins and tiny reticular veins. This is well compared to the venation of an actual Ilex lohfauensis leaf as pictured on the top right of Fig. 2. It is also observed that when the relative bending stiffness of mesophyll increases, the tiny veins gradually disappear. When the relative stiffness ratio grows as large as ${{\rm{10}}}^{-3}$, branch veins turn inconspicuous. This is compared with an India rubber fig leaf (as pictured on the bottom right of Fig. 2), whose mesophyll is relatively stiffer. It is noted that the main vein of actual India rubber fig leaf is longer than that from the optimized results when the relative stiffness ratio equals to ${{\rm{10}}}^{-3}$. This is possibly due to the non-mechanical requirement for the venation. For example, a leaf-long vein facilitates water transportation throughout the leaves.

The aforementioned optimization results provide insightful understanding of venation. It is indicated that providing structural support is a crucial function of leaf vein, especially when the mesophyll is relatively thin and soft. In the case of stiff vein and thin soft mesophyll, fractal branch veins and reticular tiny veins provide web supports for the mesophyll, which minimize the structural deformation and therefore guarantee sunlighted area of leaf. In the case of thick mesophyll, which can provide a considerably strong support to itself, a main vein with large diameter is desired, whilst narrow sub veins are no longer necessary.

### Numerical verification of the optimized results

**Figure 3:**
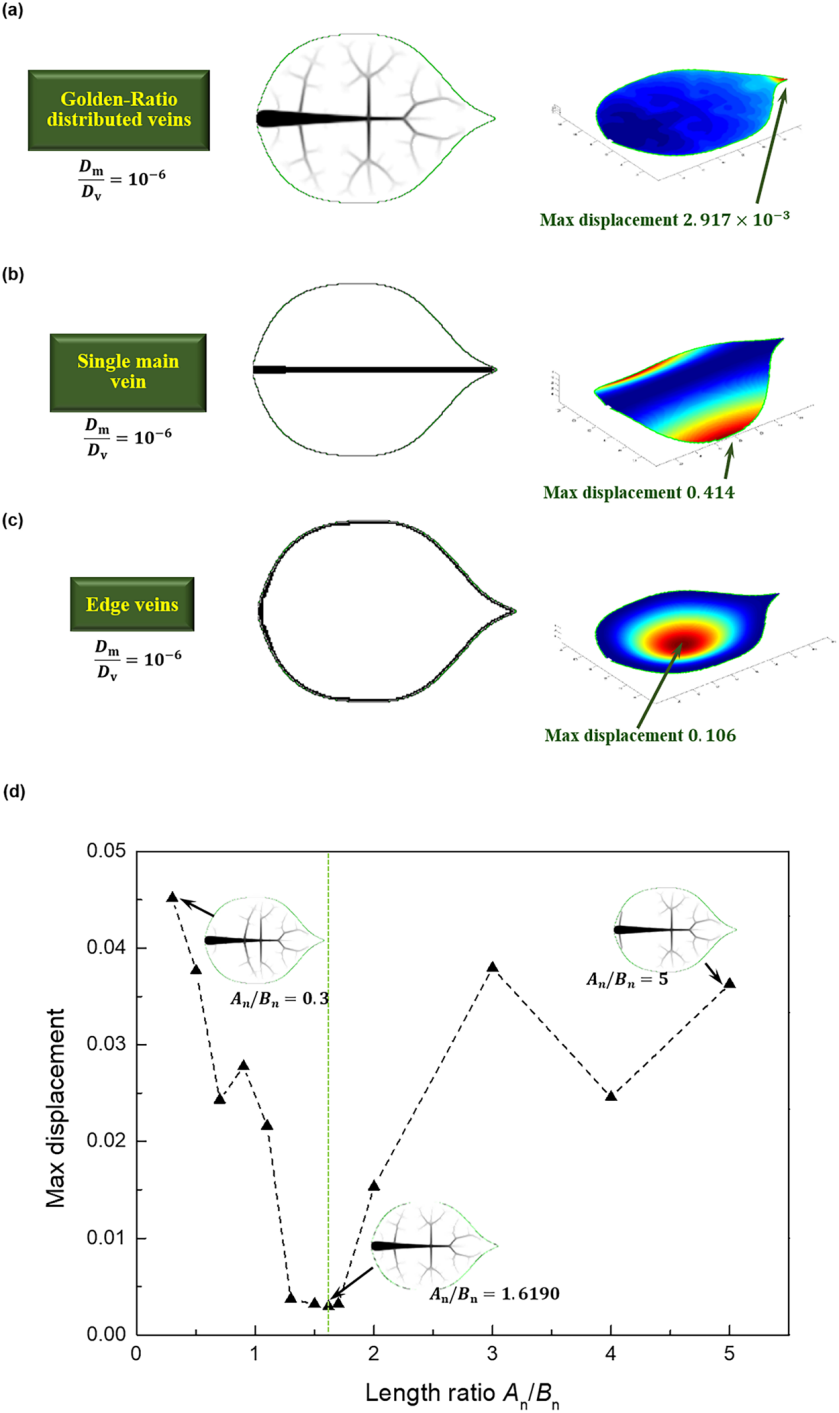
Displacement of leaves with various venations; Golden-Ratio distribution exhibits maximized structural stiffness.

The optimized results are firstly verified in this section by comparing the numerical results of maximum deformations of leaves with various venation. Three typical ovate-shaped leaves with different venations are firstly compared in Fig. 3a–c, i.e., leaf with GR distributed veins, leaf with single main vein, and leaf with edge vein. The area percentage of veins are all 5%, and the relative stiffness ratio of mesophyll are ${{\rm{10}}}^{-6}$. As shown in Fig. 3a, the leaf with GR distributed veins exhibited basically uniform displacement. Noting that the values of displacement are normalized by the total length of the leaf. The displacement at the gap between branch veins are relatively larger, while the maximum displacement of 2.917 × ${{\rm{10}}}^{-3}$ appears in the apex of leaf. For leaf with only a single main vein in the middle, although the stiff main vein with higher width can significantly reduce the displacement near the vein, namely the displacement at the apex is decreased by more than 60% to 1.083 × ${{\rm{10}}}^{-3}$, the leaf without web supports from fractal veins shows extremely large displacement. The maximum displacement of 0.414 occurs at the middle of edge of the leaf with single main vein. Figure 3c illustrates the displacement distribution of a leaf with edge vein. Similarly, the displacement of points near the vein are effectively reduced, yet the displacement of points away from the edge vein are much higher. The maximum displacement of leaf with edge vein is found appearing at the center.

Though the maximum displacement of leaf with edge vein (0.106) is 3534% higher than the maximum displacement of leaf with GR distributed veins, it is still 74% lower than the maximum displacement of leaf with single main vein. The comparison indicates the crucial effect of web supports in providing structural stiffness, and preliminary verified that the fractal veins are effective in enhancing the structural stiffness.

Further verification is conducted by varying the length ratio of second section to first section of main vein ${A}_{{\rm{n}}}/{B}_{{\rm{n}}}$. The maximum displacement of leaves with different length ratio from 0.3 to 5.0 are plotted, as shown in Fig. 3d. The overall trend of maximum displacement is a nonlinearly fluctuation with the increase of length ratio. The maximum displacement is found to be minimized (2.917 × ${{\rm{10}}}^{-3}$), when the length ratio is 1.6190, as the optimized results given. The maximum displacement is 3.167 × ${{\rm{10}}}^{-3}$, which is slightly higher than the minimum value, when the length ratio is 1.5 or 1.7. For leaf with other length ratio, the maximum displacement of are all apparently higher than the minimized values of 2.917 × ${{\rm{10}}}^{-3}$. Therefore, the numerical results verified that the GR distributed veins are optimized for maximizing the structural bending stiffness.

### Experimental verification of the optimized results

The GR distribution of leaf veins is further verified by experimental measurements of actual leaves in this section. A total of 103 ovate-shaped leaves, including magnolia denudate leaves, syringa leaves, and ilex lohfauensis leaves, are scanned to measure the length ratio of the second section to first section of main veins.

**Figure 4:**
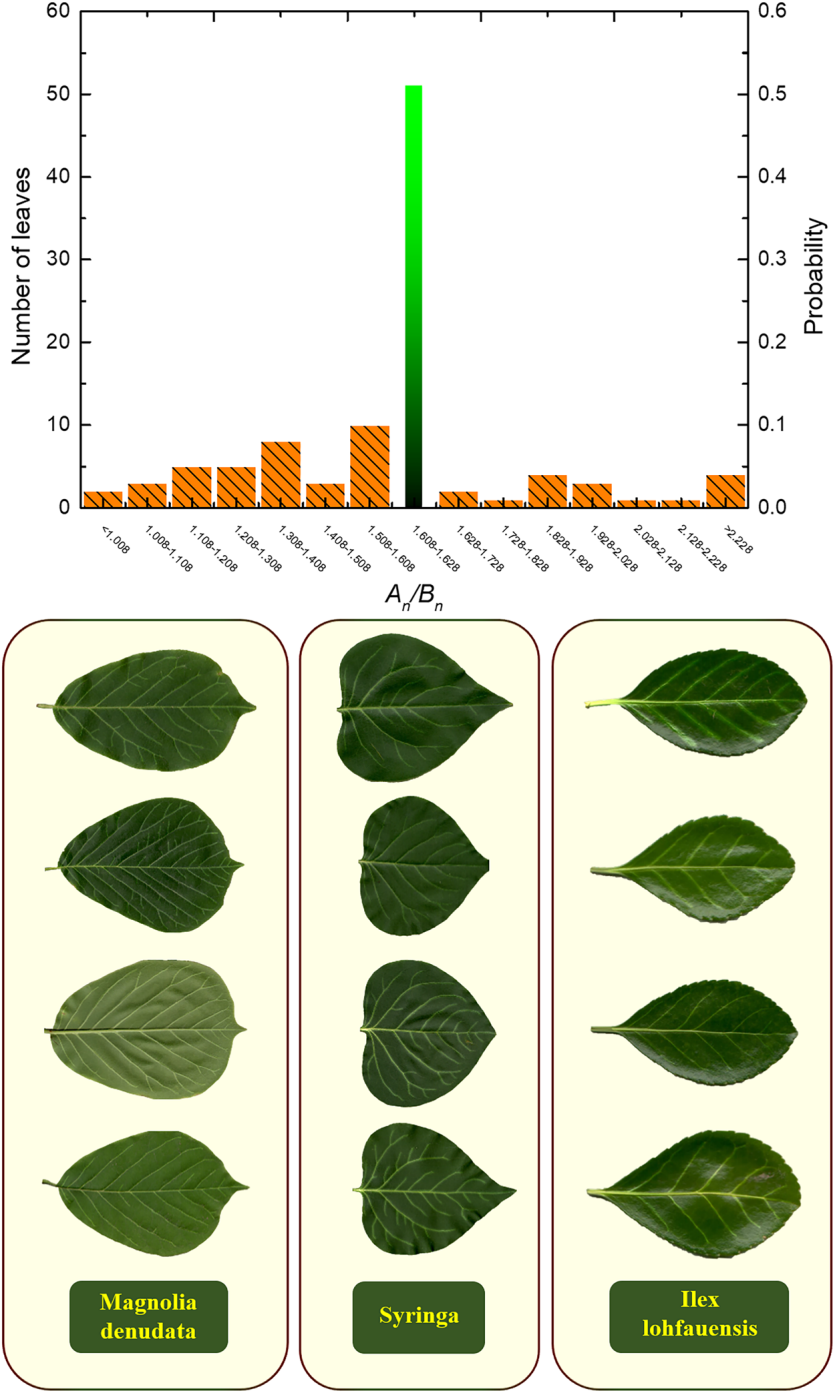
The length ratio ${A}_{{\rm{n}}}/{B}_{{\rm{n}}}$ for various leaves, and typical leaf specimens; showing that nearly a half of the natural leaves exhibit almost GR vein distribution.

Figure 4 demonstrates the statistics and typical scanned images of the leaf specimens. The average value of length ratio ${A}_{{\rm{n}}}/{B}_{{\rm{n}}}$ measurements is 1.61203, which is very close to the golden ratio (only 0.36% relatively lower). For generating Fig. 4, we divide the total sampling interval of ${A}_{{\rm{n}}}/{B}_{{\rm{n}}}$. values into several sub-intervals of size 0.1. Then the numbers of samples whose ${A}_{{\rm{n}}}/{B}_{{\rm{n}}}$ values fall in the corresponding intervals are counted. Here in order to address the accuracy of our predictions, we squeeze the size of the interval containing the GR value to 0.02, that is, [1.608, 1.628].

It is observed that nearly a half (51 out of 103) of the natural leaves exhibit almost GR vein distribution (with length ratio of 1.618 ± 0.01). This means that the probability of length ratio ${A}_{{\rm{n}}}/{B}_{{\rm{n}}}$ in the small interval [1.608, 1.628] is 49.51%. In contrast, the fractions of leaves falling in each non-GR interval are no more than 9.7%. Therefore, the average measurements of length ratio and its extensively appearance near the golden ratio suggest that the ovate-shape leaves generally tend to take a GR distributed veins. The experimental measurements in this study further verified that the GR distribution of leaf veins could provide optimized structural stiffness and sunlighted area. The GR principle may provide a helpful guideline for the design of biological tissues and biomimetic functional structures.

## Discussion

Our experiments and simulations have examined the critical role of leaf vein in providing structural support and guaranteeing sunlighted area. The results firstly indicate that GR commonly exits in the optimized distribution of vein reinforcement for maximizing the structural bending stiffness of leaves. It is suggested that leaves have evolved GR distributed veins for providing higher structural stiffness. Furthermore, it is found that the relative bending stiffness between veins and mesophyll has a significant influence on the distribution of thinner veins. Multi-level fractal veins, consisting of one thick, long, main vein and numerous thin, short, sub veins, are suggested to be the optimized configuration when the relative bending stiffness of mesophyll is low. In contrast, only the thick, long, main vein remains in the optimized configuration when the relative bending stiffness of mesophyll is high. The present study provides insight into how plants may achieve better structural performance and more sunlight exposure. The findings and uncovered mechanical principles may also be useful for future design of biomimetic functional structures^21–23^.

It is worth mentioning that the GR distribution of leaf veins is common for plants, not a must since the actual shape of a leaf is influenced by many natural conditions, including weather, water, plant diseases and insect pests. The natural conditions lead to the commonly asymmetric shape of actual leaves with asymmetric venation. It is believed that a more similar vein pattern to real leaves could be obtained by properly taking relevant factors into consideration (by imposing corresponding constraints in the problem formulations). Other parameters, including water transportation, leaf curvature, uniformity and aspect ratio, are still to be studied to fully exploit the principles behind the distribution of veins in plant leaves.

## Methods

### Stiffness measurement

Leaves of different species are freshly collected from the branches as typical leaves for stiffness measurements. The collected leaves are cleaned by water and then cut into separately vein part and mesophyll part immediately to avoid excessive loss of water. An Instron testing machine is used to test the tensile modulus of mesophyll and vein. In conjunction with the geometric measurements of leaves, the bending stiffness of mesophyll and vein can be calculated as follows

1 $$D=\frac{E{t}^{3}}{12(1-{\nu }^{2})},$$

where $D$ is bending stiffness of mesophyll or vein, $E$, *t* and *ν* are the Young’s modulus, thickness and Poisson ratio of the mesophyll or vein, respectively.

### Finite element analysis

The deformations of typical leaves under self-weight are calculated by solving plate bending problems under pressure load using Finite Element Method (FEM). Specifically, the leaf mesophyll is simplified as a thin plate, and the leaf veins are converted into equivalent reinforcements with higher bending stiffness.

In the present study, 4-node square-shape plate element is used for FE analysis. For simplicity, the bending stiffness of vein reinforcements is normalized as 1, and the bending stiffness of leaf mesophyll is normalized as ${{\rm{10}}}^{-9}$ to ${{\rm{10}}}^{-3}$, according to Table 1. The bending stiffness of mixed elements, which contain both vein part and mesophyll part, are calculated by the homogenization approach^24,25^. A normalized uniformly distributed pressure load of unit magnitude is used to represent the action of leaf weight. Clamped boundary condition is prescribed to the joint between leaf blade and leaf petiole, where all degrees of freedom are set to be fixed.

### Formulation of the optimization problem

According to the descriptions made in the main text, the topological optimization problem of maximizing the structural bending stiffness of a leaf can be formulated under the Solid Isotropic Material with Penalization (SIMP) framework^24^ as

2 $$\begin{array}{c}\,\,{\rm{Find}}\,\rho =\rho (x,y),\,W=W(x,y),\\ {\rm{Minimize}}\,C={\int }_{{\rm{\Omega }}}P(x,y)W(x,y){\rm{d}}A,\\ {\rm{s}}.\,{\rm{t}}.\,\\ \,D(\rho (x,y)){\nabla }^{4}W(x,y)=P(x,y),\,(x,y)\in {\rm{\Omega }}\\ \,\,\,\,\,\,\,\,\,A={\int }_{{\rm{\Omega }}}\rho (x,y){\rm{d}}A\le \bar{A},\\ \,\,\,\,\,\,\,\,\,0\le \rho (x,y)\le 1,\\ \,\,\,\,\,\,\,\,\,W=\bar{W},{\mathrm{on}{\rm{\Gamma }}}_{W},\end{array}$$

where $\rho (x,y)$ is a density function which represents the distribution of vein reinforcements,$\,P(x,y)$ and $W(x,y)$ are the pressured external load and deflection of the middle surface ${\rm{\Omega }}$ of the leaf, respectively, $C$ is the work of external load which is inversely proportional to structural bending stiffness, $D(\rho (x,y))={D}_{{\rm{m}}}+({D}_{{\rm{v}}}-{D}_{{\rm{m}}}){\rho }^{p}$ is the penalized bending stiffness, ${D}_{{\rm{m}}}$ and ${D}_{{\rm{v}}}$ are the bending stiffness of mesophyll and vein respectively, $p=3$ is a penalization factor. In equation (2) $\bar{A}$ is the upper bound of the area of the vein reinforcement, and $\bar{W}$ is the prescribed boundary displacement on Dirichlet boundary. The topology optimization is solved with use of the Method of Moving Asymptotes (MMA) algorithm.

### Sensitivity analysis

The MMA algorithm, which searches the optimal results based on gradient informants, requires explicit sensitivities of objective function and constraint functions. The sensitivity of the objective function, i.e., the partial derivative of the objective function with respect to the design variables, can be calculated as

3 $$\frac{\partial C}{\partial {\rho }_{i}}=-\,p({D}_{{\rm{v}}}-{D}_{{\rm{m}}}){\rho }_{i}^{p-1}{{\boldsymbol{w}}}_{i}^{\top }{{\bf{k}}}_{0}{{\boldsymbol{w}}}_{i},\,i=1,\ldots ,n,\,$$

where ${\rho }_{i}$ is the density of the *i*-th element, ${{\boldsymbol{w}}}_{i}$ is the corresponding element displacement vector, ${{\boldsymbol{k}}}_{i}$ is the element stiffness matrix when the Young’s modulus of the *i*-th element takes a unit value. In equation (3),$\,n$ is the total number of finite elements.

Similarly, the sensitivity of the constraint function in this study can be given by

4 $$\frac{{\rm{d}}A}{{\rm{d}}{\rho }_{i}}=1,\,i=1,\ldots ,n.\,$$

## Data Availability

The datasets generated during and/or analysed during the current study are available from the corresponding author on reasonable request.
